# Data model harmonization for the All Of Us Research Program: Transforming i2b2 data into the OMOP common data model

**DOI:** 10.1371/journal.pone.0212463

**Published:** 2019-02-19

**Authors:** Jeffrey G. Klann, Matthew A. H. Joss, Kevin Embree, Shawn N. Murphy

**Affiliations:** 1 Research Information Science and Computing, Partners Healthcare, Boston, Massachusetts, United States of America; 2 Harvard Medical School, Boston, Massachusetts, United States of America; 3 Laboratory of Computer Science, Department of Medicine, Massachusetts General Hospital, Boston, Massachusetts, United States of America; 4 Personalized Medicine, Partners Healthcare, Boston, Massachusetts, United States of America; Hopitaux Universitaires de Geneve, SWITZERLAND

## Abstract

**Background:**

The All Of Us Research Program (AOU) is building a nationwide cohort of one million patients’ EHR and genomic data. Data interoperability is paramount to the program’s success. AOU is standardizing its EHR data around the Observational Medical Outcomes Partnership (OMOP) data model. OMOP is one of several standard data models presently used in national-scale initiatives. Each model is unique enough to make interoperability difficult. The i2b2 data warehousing and analytics platform is used at over 200 sites worldwide, which uses a flexible ontology-driven approach for data storage. We previously demonstrated this ontology system can drive data reconfiguration, to transform data into new formats without site-specific programming. We previously implemented this on our 12-site Accessible Research Commons for Health (ARCH) network to transform i2b2 into the Patient Centered Outcomes Research Network model.

**Methods and results:**

Here, we leverage our investment in i2b2 high-performance transformations to support the AOU OMOP data pipeline. Because the ARCH ontology has gained widespread national interest (through the Accrual to Clinical Trials network, other PCORnet networks, and the Nebraska Lexicon), we leveraged sites’ existing investments into this standard ontology. We developed an i2b2-to-OMOP transformation, driven by the ARCH-OMOP ontology and the OMOP concept mapping dictionary. We demonstrated and validated our approach in the AOU New England HPO (NEHPO). First, we transformed into OMOP a fake patient dataset in i2b2 and verified through AOU tools that the data was structurally compliant with OMOP. We then transformed a subset of data in the Partners Healthcare data warehouse into OMOP. We developed a checklist of assessments to ensure the transformed data had self-integrity (e.g., the distributions have an expected shape and required fields are populated), using OMOP’s visual Achilles data quality tool. This i2b2-to-OMOP transformation is being used to send NEHPO production data to AOU. It is open-source and ready for use by other research projects.

## Introduction

The All Of Us Research Program, previously called the Precision Medicine Initiative, is a massive national undertaking to build a cohort of one million patients, who will have consented to allow access to their healthcare and genetic data for research [1]. The premise is that giving researchers access to both phenotype and genotype data on a very large, curated cohort will enable a sea change in medical research. This might speed discoveries in areas such as: individual differences in therapy response, targeted therapy development, and biomarker discovery.

The NIH describes the project as a “participant-engaged, data-driven enterprise supporting research at the intersection of lifestyle, environment, and genetics to produce new knowledge with the goal of developing more effective ways to prolong health and treat disease.”[1,2] Recruitment has been underway since the summer of 2018.

Logistically, the program is organized around a dozen Healthcare Provider Organizations (HPOs) that send their consented patients’ data to a central Data Research Center (DRC), hosted at Verily. [3,4] The data will be refreshed quarterly. In our New England HPO, patients sign up through a web portal, which stores their identity in a tracking system within the hospital. When a data refresh is requested, software extracts the medical records for all consented patients and prepares it for upload to the DRC.

Part of this preparation is converting the medical record data into a common format, that of the Observational Medical Outcomes Partnership (OMOP). This transformation is no small task, as medical data is not stored in this format or OMOP’s supported terminologies in Electronic Health Records (EHRs). OMOP is a Common Data Model (CDM) for analytics, several of which have arisen in recent years, as secondary analysis of electronic health record data has become more commonplace. Healthcare institutions tend to support at most one CDM, and the choice often depends on which national initiatives a site participates in. Each of these models have their own quirks, value sets, terminologies, and value representations, making each one unique enough to impede interoperability.

### CDM models

The CDM models presently in use by large nationwide initiatives include:

#### PCORnet common data model (PCORnet CDM)

The PCORNet Common Data Model is supported by all networks in the Patient Centered Outcomes Research Institute, and thus has a wide base of existing support. Over 80 institutions have already transformed their data into this model. [5] It was derived from the Mini-Sentinel data model, which has increasing uptake in claims data analysis.

PCORnet CDM (v3.1) is a traditional relational database design, in which each of fifteen tables corresponds to a clinical domain (e.g., diagnoses, labs, medications, etc.). The tables have many columns including both the table key (patient identifier, encounter identifier, etc.) and additional details (e.g., medication frequency). New releases of the data model have added new clinical elements or format–for example, new domains (e.g., lab values) and changes in data representation (e.g., smoking status).

#### Informatics for integrating biology in the bedside

i2b2 was first developed over a decade ago through a National Institutes of Health (NIH) grant and continues to grow in popularity. It is currently used at over 200 sites world-wide, and it is used in several large-scale networks, including the NCATS’ national Accrual to Clinical Trials (ACT) network. [6,7]

i2b2 uses a star-schema format, pioneered by General Mills in the 1970s and widely used in retail data warehouses. [8] The i2b2 star-schema uses one large “fact” table containing individual observations. This is a narrow table with many rows per patient encounter. Ontology tables (hierarchical arrangements of concepts) provide a window into the data; these are often developed by local implementers. Consequently, the data model is only modified when core features are added to the platform.

#### Observational medical outcomes partnership (OMOP)

OMOP was developed to be a shared analytics model from the beginning, and it has been adopted by the Observational Health Data Sciences and Informatics (OHDSI) Consortium, a diverse collaborative dedicated to research and quality improvement. [9] The OMOP CDM is increasingly utilized, presently at 90 sites worldwide, thanks to OHDSI’s large community and many analytical tools.

OMOP is a hybrid model that provides domain tables in the vein of PCORnet, as well as a “fact” table containing individual atomic observations similar to i2b2. The OMOP schema is significantly more complicated than PCORnet, and some domain tables are derived values for specific analytical purposes (e.g., drug_era and visit_cost). Unlike PCORnet (but similar to i2b2’s ontology system), OMOP provides metadata tables providing information on terminology and concept relationships.

Increasingly, in order to participate in multiple national initiatives, sites must support all three data models.

### High-performance data transformations

i2b2’s data model is designed to be highly adaptable and able to easily ingest data from various source systems without data transformation. Import of new types of data elements can be done directly into the fact table, and the ontology can be modified to make these data accessible to researchers.

In our previous work, we developed a “PCORnet Information Model” in i2b2, modeled as an i2b2 ontology, that exactly represents the data structure and permissible data elements of PCORnet CDM. [10] Local sites adopt this ontology and use our mapping methodology to “redirect” ontology elements to the sites’ local codes, without modifying their underlying data. Then data is transformed “on-the-fly” through the ontology module when it is queried using i2b2 or the multi-site i2b2 query system, the Shared Health Research Informatics Network (SHRINE) tool. [11]

Our PCORnet Information Model is now called the ARCH Ontology, named after our PCORnet-funded network from which it originated, the Accessible Research Commons for Health (ARCH). It has been adapted by several other large projects. The ACT network’s ontology builds from the ARCH Ontology, using the same basic terminology trees and adding some additional elements (e.g., length of stay and vital status). Several other i2b2-based PCORnet networks use variations of the ARCH Ontology. University of Nebraska Medical Center has forked the ontology and is updating its terminology trees as a primary mode of demonstrating its tools for deploying terminology standards. [12,13]

We also previously developed a high-performance data transformation that materializes the PCORnet CDM through a SQL program which is driven by the ARCH ontology. [10] The ARCH network is using this ontology and transformation to participate in both PCORnet queries and ARCH queries at 10 sites nation-wide.

**Fig 1:**
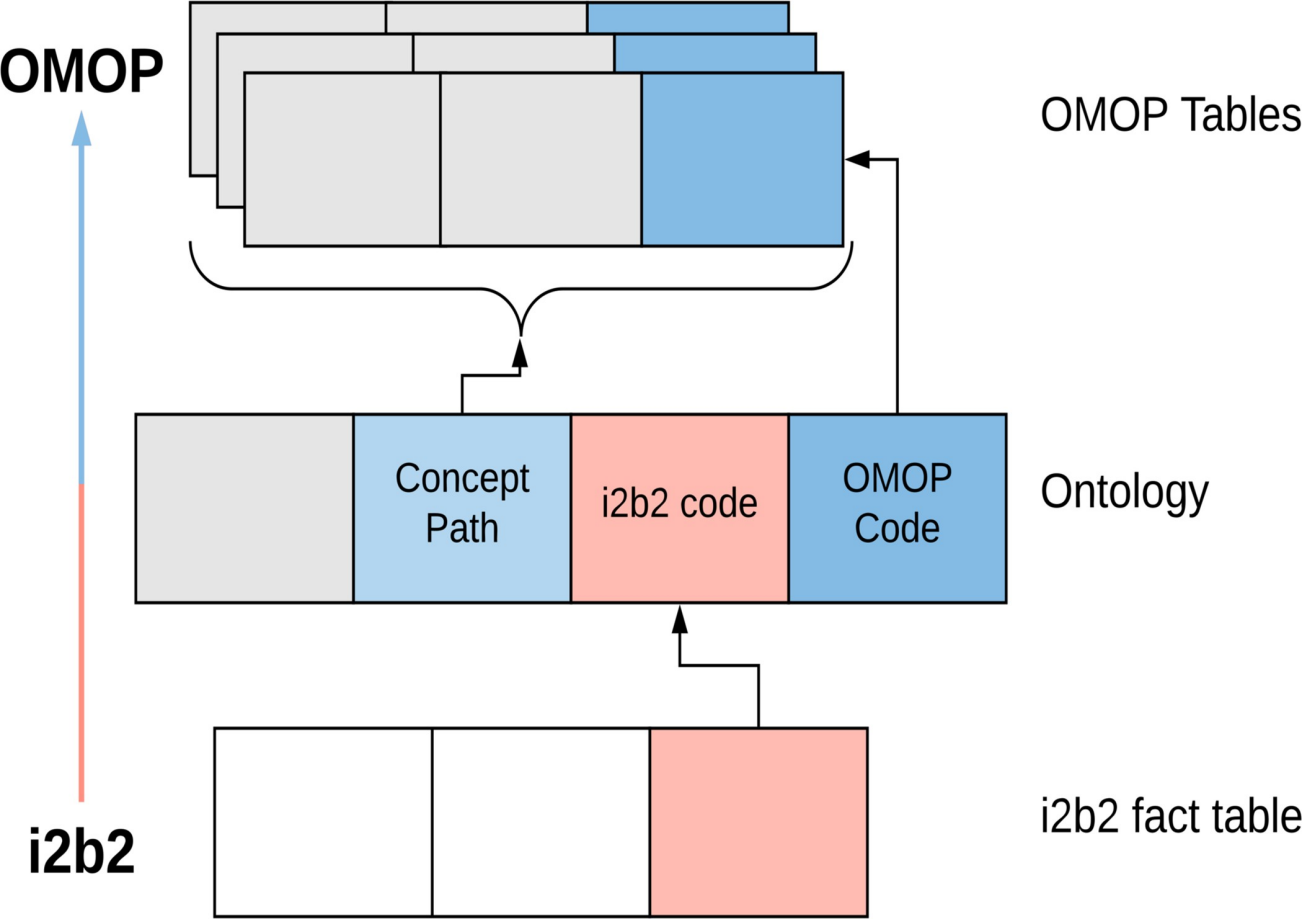
Ontology-driven data transformation in i2b2. The ontology, which defines concept metadata, drives the transformation from i2b2 to OMOP. Data are retrieved from the i2b2 fact table, converted to OMOP codes via ontology lookups, and then written to the OMOP tables specified through the ontology concept path.

Here, we apply the same methodology to develop a transformation from i2b2 to OMOP for All Of Us. This process is presented visually in Fig 1. Because of the national interest in variations of the ARCH ontology, and because our HPO’s two sites (Boston Medical Center and Partners Healthcare) had already invested significantly in mapping to the ARCH ontology, we utilized that ontology and mapping as the template for transforming data for All Of Us.

#### Objective

We are participating in All Of Us through the New England Precision Medicine Consortium, which requires us to provide quarterly data structured in OMOP. Our sites maintain active primary i2b2 repositories for internal research.

Our sites also participate in PCORnet through the Accessible Research Commons For Health (ARCH) subnetwork, which uses the widely adopted ARCH ontology based on the PCORnet CDM. Our sites use a previously-published methodology and information model to transform i2b2 data into PCORNet CDM. [10]

Here we describe our unique solution, which leverages our previous expertise in information-model-driven i2b2 data transformations to transform i2b2 data directly into OMOP. This work has also given us a detailed understanding of the differences and similarities among these three data models.

## Methods

i2b2 ontologies can represent “Information Models” which describe the exact information (data elements, data types, and codes) that can be encoded in a corresponding data model. For example, our ARCH ontology provides (among other terminologies) hierarchies of all available diagnosis codes, in ICD-9 and ICD-10 format. This information model then drives the transformation of those data elements into the table format of the target data model, using the hierarchical nature of the ontology to group local codes into standard codes. For example, “PHS_DIAB” and “PHS_DIABETES” might be arranged as children of the standardized code “ICD9:250”, that will be inserted into the target data table.

This is discussed in more detail in our previous paper, in which we demonstrated that this methodology could scale well for the PCORnet CDM. [10] We continue to maintain an ARCH ontology and transform into the PCORnet CDM.

All Of Us presented us with the opportunity to explore data transformation into the more complex OMOP CDM.

We examined the differences between OMOP and PCORnet CDM to ascertain the potential difficulties in developing a new data transformation. In the following analysis, PCORnet CDM refers to v3.1, the latest widely-implemented version, the ARCH ontology refers to v3.1b, also based on PCORnet CDM v3.1, and OMOP refers to All Of Us’ subset of OMOP 5.1. This subset defines tables that are considered the most clinically relevant domains and excludes fields deemed to be low priority. [14]

### OMOP data model comparison

#### Terminology

OMOP tables appear to have an exact correspondence to our ARCH Information Model. However, detailed analysis reveals an important difference.

Most i2b2 ontologies (including the ARCH Information Model, the i2b2 demo ontology, and ontologies for other i2b2 networks like ACT) derive the majority of their content from data provided by standards organizations. For example, to build the ARCH ontology, we extensively used a tool which builds i2b2 hierarchies from BioPortal. [15]

OMOP maintains its own terminology dictionary, consisting of over 3 million terms from 43 standard terminologies. However, OHDSI modifies the terminologies after receiving them from standards organizations, reassigning some terms to different target tables. This assignment is done by terminology experts who are attempting to correct weaknesses in the source terminology, and to improve the data’s utility for analytics. They dub these poorly assigned terms ‘dirty.’ [16] For example, OMOP assigns over 1300 ICD diagnosis codes to the procedure table. Some of these are vaccines, which are diagnosis codes for historical reasons but analytically are procedures, so OMOP assigns them to the procedure table.

Also, OMOP requires data in a set of the most analytically complete terminologies, which are optimal for data analysis (LOINC, SNOMED, and RxNorm). Our ARCH ontology uses trees recommended by PCORnet, which uses terminologies actually seen in billing and EHR data (e.g., ICD, NDC, and CPT).

Table 1 shows our ARCH ontology hierarchies and terminologies, and their equivalents in OMOP.

**Table 1:** ARCH ontologies and terminologies vs OMOP.

i2b2 ARCH ontology tree	ARCH terminology provided	OMOP Table	OMOP Terminology	PCORnet Equivalent Table
**Encounter**	*PCORnet valueset*	Visit Occurrence	*OMOP valueset*	Encounter
**Demographics**	*PCORnet valueset*	Person	*OMOP valueset*	Demographics
**Diagnosis**	ICD-9, ICD-10	Condition Occurrence, Measurement, Procedure Occurrence, Observation	SNOMED	Diagnoses AND Condition
**Procedure**	ICD-9, ICD-10, CPT, HCPCS	Procedure Occurrence, Device Exposure, Drug Exposure, Observation	S*ame as i2b2 terminologies*	Procedure
**Labs**	LOINC	Measurement	LOINC	Lab_Result_CM
**Vitals**	*PCORnet valueset*	Measurement	LOINC	Vitals
**Medication**	RxNorm, NDC	Drug Exposure	RxNorm	Prescribing AND Dispensing

#### OMOP modifiers comparison

The i2b2 fact table provides details about the core facts in a patient encounter, such as “Prescription for Zantac.” Ancillary data that provides additional detail about a parent fact, such as “Twice daily,” we call *modifiers*. Relational data models like PCORnet and OMOP CDM tend to have many of these, because they only involve adding additional columns to the data model. In our experience, these additional modifiers tend not to be available in data warehouses, so the result is many empty columns. i2b2 supports modifiers through the ontology system and a special modifier code field in the fact table. Multiple modifiers can be expressed by duplicating a fact and changing only the modifier.

Modifiers tend not to be standardized across information models. We compared the modifiers implemented in the ARCH ontology to those supported by OMOP, and we found that only three (medication refill, quantity, and supply) are the same between approaches. Four more are present in both but use different value sets. For example, ARCH distinguishes between prescribed, dispensed, and administered drugs, but not the subtler variations supported by OMOP, such as “dispensed through mail order” or “Physician administered drug, identified from referral record.” The remaining 18 modifiers described by OMOP or PCORnet are present in only one model (though the majority of these are detailed medication data). Our analysis of modifiers can be seen in Table 2.

**Table 2:** ARCH Ontology modifiers vs. those in OMOP.

Domain	Modifier	ARCH	OMOP
Diagnosis	Condition vs Diagnosis		
	Primary/Secondary		
	Stop Reason		
Procedure	Primary/Secondary		
Laboratory	Lab Priority		
	Lab Location		
	Prescribing vs. Dispensed		
Medication	Refills		
	Quantity		
	Supply		
	Dose, Route, Sig, Stop Reason, Lot #, effective drug dose; route concept id; sig; stop reason; lot #		
	Frequency		
Vitals	Source		
	Position		
	Normal Range		

Yellow: terminological differences. Red: not present. Green: equivalence between models.

#### The OMOP transformation

Next, we defined a new information model and data transformation from i2b2 into OMOP. We focused on achieving interoperability between data transformations and minimizing the additional work needed for sites to transform their data from i2b2 into OMOP, by reusing the ARCH ontology as much as possible. (Our sites and others are already using and have mapped their data to the ARCH ontology.)

We elected to reuse the widely-adopted and EHR-centric terminology trees in the ARCH ontology, mapping data to OMOP terminologies and reassigning target tables during transformation. Because in our experience modifiers are very infrequently implemented, we also elected to initially offer only the eight ARCH modifiers that could be mapped to OMOP.

Finally, we tested our results by running the Achilles data characterization tool on a subset of OMOP data and validating that the expected fields were populated, and the data distribution looked as expected.

## Results

### Transformation implementation

We developed and deployed an ARCH-OMOP ontology derived from the ARCH ontology, but with an important change. OMOP version 5 assigns its own unique "OMOP numbers" to every term. For example, ICD-9 code 250 (diabetes mellitus) is 44833365 in OMOP. To support this, we added an *OMOP code* column to our information model with a direct cross-reference to the OMOP number in the concept dictionary. (We also disabled both incompatible modifiers and HCPCS codes, which are included for demonstration purposes but not used in ARCH because our HCPCS hierarchy is very outdated.)

A small number of codes in our vocabulary trees did not appear in the OMOP concept dictionary: 102 RxNorm codes, 10 NDC codes, 126 ICD-9 codes, and 11 CPT procedure codes. Most of these codes are archaic or non-standard–e.g., over-the-counter meds and temporary billing codes. To assess their use, we analyzed their appearance in the entire ARCH network. Only 8 of these codes appear at all. These are shown in Table 3.

**Table 3:** Codes that could not be found in the OMOP concept dictionary.

Code Type	Name	Code	~# pts
**RxNorm**	Benzocaine/menthol	466426	50,000
**CPT**	(retired) Antibody, non-RBC, quantitative, first antigen	86008	2,000
**RxNorm**	Citric acid/simethicone/sodium bicarbonate	689842	2,000
**RxNorm**	Acetaminophen/diphenhydramine/pseudoephedrine	689786	1,000
**CPT**	Abdominal pain, unspecified site	78900	1,000
**CPT**	Individual medical psychotherapy by a physician…	90841–90844	500
**CPT**	Hepatitis C antibody	86302	200
**RxNorm**	Fentanyl citrate, 0.05 mg/ml injectable solution	856409	100
**CPT**	(retired) ADP Titration Platelet Aggregation Study	85575	100
**CPT**	(retired) Kidney function study including pharmacologic intervention	78726	100

Approximate term frequency in ARCH is shown to the right, as a measure of the data loss caused by the missing code.

We next developed a SQL program that would utilize the ARCH-OMOP ontology mappings to instantiate OMOP tables. The SQL script is similar to the one we previously developed for PCORnet–it copies data from i2b2 one table at a time into OMOP. The transformation is driven by the ontology, looking up each i2b2 code in the ontology and writing the equivalent OMOP number to the correct OMOP table and column. The list of source tables/ontologies to target tables is shown in Table 4. This methodology is visually depicted in Fig 1 and described in more detail in our previous work.

**Table 4:** Transformation source to target table.

i2b2	OMOP
**No mapping–Table copy directly from i2b2**	
**Visit Dimension**	Care Site
**Patient Dimension**	Death
**Provider Dimension**	Provider
**Mappings derived from OMOP Concept table**	
**ARCH Diagnosis**	Condition Occurrence, Observation, Device, Measurement, Procedure
**ARCH Procedure**	Procedure, Observation, Measurement, Condition, Drug Exposure
**ARCH Medications**	Prescribing
**Mappings provided in the All Of Us Specification**	
**ARCH Demographics**	Person
**ARCH Encounter**	Visit Occurrence
**ARCH Vitals**	Measurement
**Computed Tables**	
**Visit Dimension**	Observation Period
**Drug Exposure**	Drug Era
**Condition Occurrence**	Condition Era

This table is divided into four sections, showing the different ways target values are generated.

In this transform, we also use the OMOP concept dictionary to dynamically map OMOP numbers to their equivalent “standard” terminology number, using the OMOP concept_relationship table, as seen in Table 1. We map dynamically at runtime in part to automatically adapt to changes in mapping or code changes in standard terminologies, but also because the mapping is 1:n. Frequently source terms map to multiple target terms. Among all terms we support in the current transformation, across all domains, 14,000 split into two to five new terms. Some of these map to multiple target terms *in multiple domains*.

Our program therefore runs in multiple passes, once for each pair of source domains and target tables. It dynamically computes the OMOP number to preferred OMOP code(s), so that newer mappings are automatically integrated. For example, given the ICD-9 code for childhood obesity in i2b2’s diagnosis domain, the diagnosis transform writes the SNOMED for childhood obesity to the condition table and also makes a pass through the observation table to also write a code for “weight greater than 97th percentile.”

**Fig 2:**
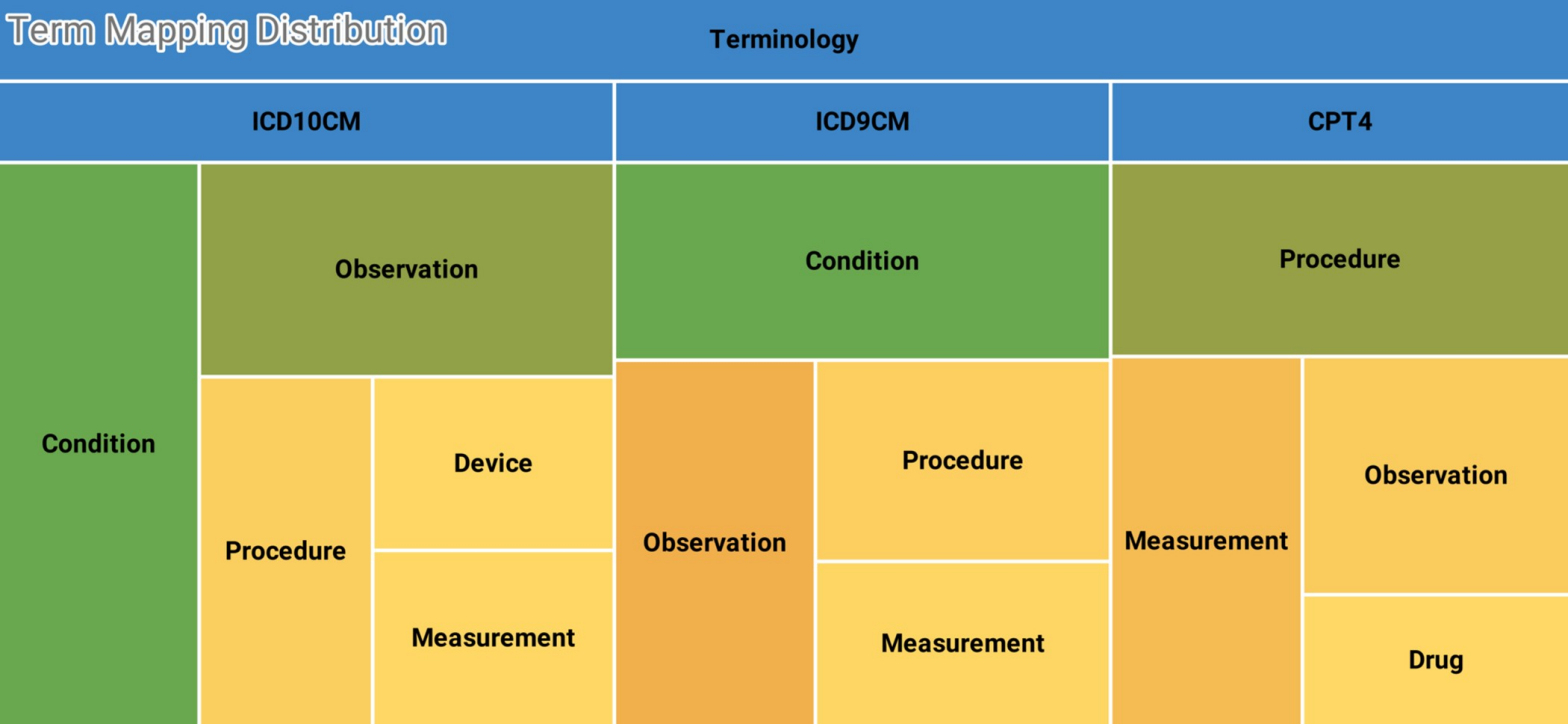
Mapping distribution from ARCH terminologies to OMOP. ICD and CPT codes map to six different tables in OMOP. This is just one (easily visualizable) aspect of the many complexities encountered in mapping. Boxes in the treemap are sized in a logarithmic scale.

Fig 2 shows a heat map illustrating this mapping multiplicity. Here we see that although ICD diagnosis codes map primarily to codes in the condition table, there are five additional tables that OMOP might specify as the target table.

### Implementation in the New England Precision Medicine Consortium

We first deployed our i2b2-to-OMOP transform (information model and SQL scripts) against the i2b2 demo database of 133 fake patients, for initial testing. The OMOP version of this 133-fake-patient database was sent to the All Of Us DRC at Columbia. They verified its integrity using their validation tools, available for download from their Github repository. [17] Our data passed all integrity tests.

For further validation, we ran the transform against a 10% sample of all patients at Partners Healthcare. On our 10% sample, we ran Achilles and Achilles Heel, standard OMOP analytical tools on which the DRC’s tools are based. Achilles’ report highlighted several data quality issues that we were able to correct. Based on our experience, we are developing a QA process using the results of this analysis. We run Achilles at every data refresh and use the report to perform additional checks. Our initial set of checks include:

- **Data Density:** All lines in the report should be increasing and be of the same magnitude. The exception is observation_period, which has one entry per patient.
- **Dashboard Report, Age of First Observation:** Should have a peak in the 20s or 30s, decreasing gradually. Age 0 will have a large spike because many babies are born that do not have follow-up care.
- **Condition, measurement, drug, procedure treemaps:** Should be populated with a variety of data (many boxes).
- **Person report:** Male/female ratio is ~50/50 and that all races are represented.
- **Data runs to the refresh date.** Each visit type in the visit report should show a graph ending close to the data refresh date.

**Fig 3:**
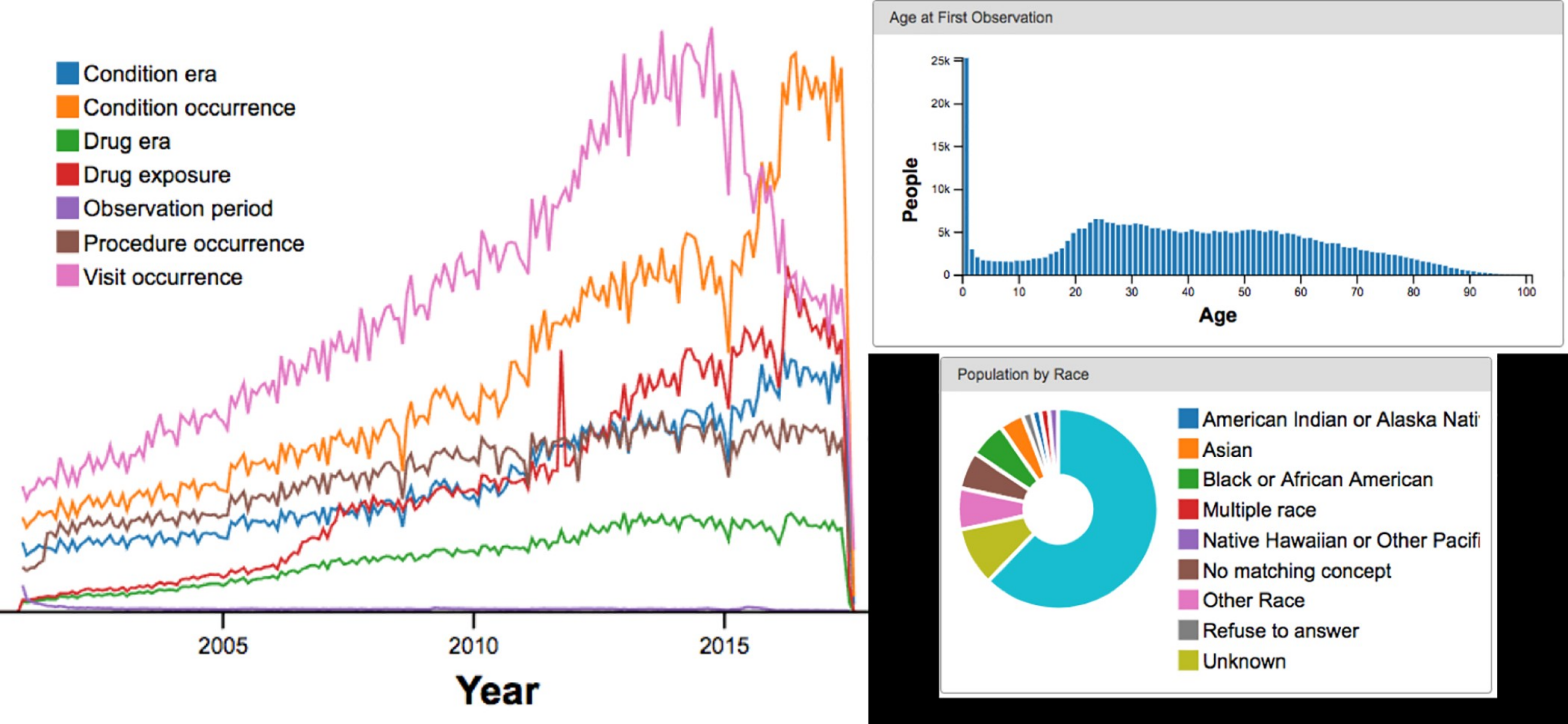
Achilles results on our “10% of Partners’ data” dataset. From top left to bottom right: (a) data density (notice all are in the same magnitude); (b) age at first observation (notice the expected peak in 20s followed by decrease, with a spike at age 0 representing babies born in the hospital but not receiving follow-up care); (c) population distribution by race.

Achilles results on our final extract of 10% of Partners data, for checks (a), (b), and (d), can be seen in Fig 3.

As of this writing, we are preparing to submit our second extract of real patient data, consisting of 7920 patient records. Boston Medical Center is also preparing their second data extract, consisting of 2809 patients’ data.

Our transformation is freely available for download from GitHub. [18] It requires the latest release of the ARCH Ontology, also available from GitHub. [19]

## Discussion/Conclusion

The All Of Us research program is a massive undertaking that encompasses all aspects of medical research, and as such it is easy to overlook the hidden complexities revolving around the data. All Of Us requires data in the OMOP analytical data model, but even among the minority of healthcare institutions that have implemented standards-based data warehouses, the majority use non-OMOP structures such as i2b2 or PCORnet CDM.

We hypothesized that our previous approach for high-capacity i2b2 data transformations would support transformation of data from i2b2 into OMOP. Due to the large national uptake of ARCH ontology variants, we based our transformation on the ARCH ontology. However, there are significant differences in terminology. These are, in our experience, the most daunting and challenging aspect of data transformations in medical informatics, and this proved true here as well. Not all terms were mappable to OMOP, and many mapped to multiple terms in multiple domains. Furthermore, because OMOP concept IDs do not match the term they represent (for example, ‘ICD-9 463’ is ‘44821975’), this makes visual inspection impossible, which complicated our testing.

The reason for these non-trivial differences between approaches is likely that analytical data models are optimized for particular use-cases. [10] OMOP, more than other approaches, focuses on robust and internationally-applicable terminologies suitable for direct analytics. The i2b2-ARCH ontology emphasizes simplicity of ingesting data, using the familiar terminologies often found in the source systems. PCORnet CDM is similar but focuses more on billing terminologies due to its heritage (a predecessor, Mini-Sentinel, is designed for claims data). Transforming between models, rather than selecting a single one, is becoming necessary to support the myriad of present use-cases in the field, so these complexities must be addressed.

Our current transform passes all criteria for initial data analysis in the All Of Us research project, and it is being used in production for All Of Us in the New England HPO. This HPO currently has 10,729 fully enrolled participants (meaning they have contributed both EHR and genetic data), and recruitment has only begun recently. It is also being utilized in several other initiatives, including an eMerge OMOP supplement. Our work will allow for considerably easier participation in OMOP-oriented initiatives. Partners Healthcare has data for 3.5 million patient lives mapped to the ARCH information model and BMC has 1.5 million. Together, they could potentially bring 5 million patient lives to the OMOP CDM.

This work demonstrates our mapping and transformation methodology is robust and reusable and that it can be applied to the analytical model required by All Of Us. Some of this success is due to the flexible design of i2b2. It supports diverse source data and is dynamically adaptable to new data element types and concepts, even when they are not represented in a standard terminology. This makes it highly adaptable to new use cases, from additional target data models to computational phenotyping algorithms.

### Limitations: OMOP

OMOP makes some unconventional decisions about data mapping that could complicate analysis if the models are not understood in some detail by researchers.

First, OMOP’s decision to split single codes into all possible target codes could potentially lead to loss of semantic precision. For example, ICD9 V37.0 (‘Other multiple, mates liveborn and stillborn, born in hospital’) maps to three SNOMED codes, for ‘multiple birth’, ‘live birth’, and ‘stillbirth’. A search for ‘live birth’ in the above case will lead to potentially false positive results, because the source term is about multiple births. Therefore, this mapping introduces potentially error-prone inference about the meaning of the term at the data transformation level.

Second, because OMOP places some codes in different tables than standard terminology trees (e.g., vaccines in diagnosis vs. procedure), queries formulated in i2b2 vs OMOP might show different results in each. This needs to be understood by the researcher or they could be misled.

### Limitations: Transformation

Our transform is robust and enterprise-ready, with the following caveats:

- We did not implement all OMOP modifiers in the ARCH ontology, and two that we did implement had incomplete value sets.
- Although validations like Achilles Heel and Achilles were run on our data, they will only tell us about the correctness of the data and not whether what is expressed in the data are useful for research.
- We only support the tables and columns required by All Of Us. The program expects to expand to include more OMOP tables in the future, so we will need to extend our transformation.
- Our SQL programs presently run only on Microsoft SQL Server. We have versions of our earlier PCORnet transform for other RDBMS platforms, so we know it is straightforward to translate the code, but we do not have the resources at present to perform this work.

### Conclusion

The complexity of data transformation is easy to overlook, especially in such a vast and complex project as the All Of Us research program. All Of Us requires data in OMOP format, although many data warehouses are organized in some other fashion, such as using i2b2. This is the case in our New England HPO. We were able to adapt our previous methodology for high-throughput i2b2 data transformation to develop an automated method to materialize OMOP tables from our data. In the process, we discovered commonalities in analytical data models, with some divergences around terminology and mapping.
